# Retraction Notice: “Adverse events after first and second doses of COVID-19 vaccination in England: a national vaccine surveillance platform self-controlled case series study”

**DOI:** 10.1177/01410768251316750

**Published:** 2025-02-18

Tsang RS, Agrawal U, Joy M, et al. Adverse events after first and second doses of COVID-19 vaccination in England: a national vaccine surveillance platform self-controlled case series study. *J R Soc Med* 2024; 117(4): 134–148. DOI: 10.1177/01410768231205430

Following the publication of this article, the journal editor was contacted by the corresponding author about duplicate publication in the *Eurosurveillance* journal.

The authors have clarified that there was no intention to submit a duplicate publication.

Due to this dual publication, the journal editor and Sage retract this article from the *Journal of the Royal Society of Medicine*.

The authors agree with the retraction of this article.

